# Cancer Pain Management and Pain Interference with Daily Functioning among Cancer Patients in Gondar University Hospital

**DOI:** 10.1155/2017/5698640

**Published:** 2017-06-12

**Authors:** Henok Getachew Tegegn, Eyob Alemayehu Gebreyohannes

**Affiliations:** Department of Clinical Pharmacy, University of Gondar, Gondar, Amhara, Ethiopia

## Abstract

Cancer is an increasing public health burden for Ethiopia. Pain is among the most common symptoms in patients with cancer. Hence, we aimed to assess cancer pain prevalence, cancer pain interference, and adequacy of cancer pain treatment in the oncology ward of an Ethiopian teaching hospital. Of 83 patients, total of 76 (91.6%) cancer patients experienced pain with varying degree of severity, and 7 (8.4%) patients experienced severe pain. Of the 76 cancer patients with pain, 68 (89.2%) experienced pain interference with their daily activities. Fifty-four (65%) patients were receiving inadequate cancer pain treatment with negative Pain Management Index. Therefore, it is vital to anticipate and assess pain of the cancer patients as routine clinical practice, to optimize analgesic therapy, and to identify and overcome barriers to adequate pain management.

## 1. Introduction

Cancer is an increasing public health burden for Ethiopia and currently accounts for four per cent of all deaths in Ethiopia [1]. Most cancer patients experience pain as one of the most common symptoms due to either the cancer itself (the primary tumor or metastases) and/or the cancer treatment (surgical, chemotherapy, radiotherapy, and others) [2, 3]. However, information about the prevalence of cancer pain in other parts of Ethiopia is limited.

Cancer pain is more common in patients with advanced or metastatic cancer [4, 5]. Nearly half of cancer patients report interference in daily activity caused by pain [6, 7]. Pain, even when treated, is often severe enough to impair their ability to function [8].

Despite availability of several established guidelines for the management of cancer pain, many cancer patients frequently receive inadequate pain treatment and undertreatment is well documented [2, 4, 9]. Patients often impede their own treatment due to misconceptions about analgesics and their side-effects, nonadherence to treatment regimens, and poor communication of their pain and their concerns about pain to health care providers. Other barriers include inadequate assessment of pain and pain management, patients' reluctance to report their pain or to give a pain score, and inadequate knowledge of pain management of professionals [10].

GUH (Gondar University Hospital) has recently (2014) opened an oncology ward. The severity and adequacy of cancer pain management in GUH are unknown and this study will help understand the severity and adequacy of cancer pain and its interference on their daily functioning of such patients in this hospital. Hence, we aimed to assess pain interference on patients' daily functioning and associated contributing factors and adequacy of cancer pain treatment in the oncology ward of GUH.

## 2. Methods

The study was conducted from February 15 to May 15, 2016, in the oncology ward of GUH, Gondar, north-west Ethiopia. GUH is a teaching and referral hospital located in the northwest Ethiopia 727 kilo meters from the capital Addis Ababa. The hospital serves an estimated 7 million people and the oncology ward is providing service with ten beds.

Patients were considered eligible if they fulfill the following criteria: age 18 years and older; diagnosed with any type of cancer; and admitted to the oncology ward in the specified time period. However, patients with other medical or psychiatric problems and unable or unwilling to provide the required information were excluded from the study.

Before conducting the study, ethical clearance was secured from the University of Gondar School of Pharmacy ethical review committee and permission to collect data from patients was obtained from Gondar University Hospital. Then, after explaining the anonymity of the interview, verbal informed consent was obtained from the respondents (patients) for the interview. Furthermore, the data collected from each patient was kept confidential and used strictly only for the purpose of the study.

A questionnaire-based interview using Brief Pain Inventory-Short Form (BPI-sf) [11] and chart review were used as data collection procedures. BPI-sf is an 8-item self-administered questionnaire used to evaluate the severity of a patient's pain and the impact of this pain on the patient's daily functioning. Sociodemographic characteristics and clinical data including patient diagnoses, comorbidity, sites of cancer, tumor stages, presence of metastases, history of cancer treatment modality, and number of medications prescribed and analgesics prescribed were gathered. Comorbidity in this study refers to the presence of one or more additional diseases or disorders cooccurring with the primary diagnosis (cancer in our study). The questionnaire was first translated into Amharic by the one of the investigators (HGT) and then back translated to English by the other investigator (EAG) to verify accuracy. This was finally translated again to Amharic (see Appendix). Data regarding the patients' medical conditions and types of analgesics prescribed were obtained from the chart review. These pieces of information were supplemented by interview of health professionals (nurses and doctors) for some variables like lists of drugs prescribed during hospital stay, tumor stage, and the presence of metastasis. Patients were interviewed regarding type of pain with its grading, any analgesic use with percentage of pain relief, and pain interference with routine life processes using BPI-sf. BPI-sf had 8 items. Item number 1 helps identify areas where patients feel pain. Item numbers 2 to 5 measure pain severity. Then,* pain severity score was* calculated by adding the scores for items 2, 3, 4, and 5 and then dividing by 4 [12]. This gives a severity score out of 10. Item numbers 6 and 7 described types of medications used for pain management and how much pain relief patients got in terms of percentage. Item numbers 8 (8.1 to 8.7) measured how much pain has interfered with seven daily activities, including general activity, walking, work, mood, enjoyment of life, relations with others, and sleep. The interference items were presented with 0–10 scales, with 0 = no interference and 10 = interferes completely. Pain interference score was calculated by adding the scores for questions 8.1, 8.2, 8.3, 8.4, 8.5, 8.6, and 8.7 and then dividing by 7. This gives an interference score out of 10. Depending on the intensity of pain, both pain severity and pain interference were classified, using BPI-sf, into four groups: no pain (0), mild pain (1 to 3), moderate pain (4 to 7), and severe pain (8 to 10).

Based on the type of analgesic medication(s) the patients were receiving, the following scores were given: 0 (no analgesic medication), 1 (a nonopioid analgesic medication), 2 (a weak opioid analgesic medication), and 3 (a strong opioid analgesic medication). Subsequently we determined the Pain Management Index (PMI). To construct PMI, the 4 levels of analgesic drug therapy used were determined by the potency: (0) no order for analgesic, (1) nonopioid (e.g., NSAID or acetaminophen), (2) weak opioid (e.g., codeine), and (3) strong opioid (e.g., morphine). Potency of analgesic was then compared with “pain worst.” Absence of pain was scored as “0,” mild pain as “1,” moderate pain as “2,” and severe pain as “3.” The PMI is computed by subtracting the pain level from the analgesic level. It ranges in value from −3 (a patient with severe pain receiving no analgesic drugs) to +3 (the patient receiving morphine or an equivalent and reporting no pain). A negative PMI score was considered an indicator of potentially inadequate pain management by the prescriber.

All statistical analysis was performed using IBM SPSS ver. 22 (IBM Co., Armonk, NY, USA). Descriptive statistics were used to summarize demographic characteristics, sites of cancer, tumor stages, presence of metastases, history of cancer treatment modality, history and type of pain, number of medications prescribed, and types and number of analgesics prescribed. Association between predictive variables (demographic data of patients) and outcome of interests (pain management adequacy and pain interference on functioning) using binary logistic regression and Fisher's exact test was done to identify determinants of outcome of interest. According to Hosmer-Lemeshow assumption, variables with a *P* value < 0.2 in a univariate analysis were included to the final model of multivariate logistic regression analysis. Multivariate analysis was performed to compute adjusted odds ratio (AOR). Statistical significance was set at a one-sided *P* value < 0.05 in the multivariate analysis.

## 3. Results

A total of 83 cancer patients, meeting the inclusion criteria, were identified during the 5-month study period between January and May 2016 in Oncology Ward of Gondar University Hospital. The median age of the patients was 50 ranging from 18 to 72 years; 50.6% were male. Higher proportions of patients with cancer were admitted in the Oncology Department due to genitourinary cancer (25.3%) and gastrointestinal cancer (22.9%). Most of the cancer patients had metastasis (73.5%), with stage 4 tumor observed in 19/83 (22.9%) cancer patients (Table 1). No analgesics were prescribed in 50 (60.2%) patients. A total of 71 (85.5%) cancer patients have ever experienced pain previously. Nociceptive pain was observed in 33/76 (43.4%) cancer patients (Table 1).

**Figure 1:**
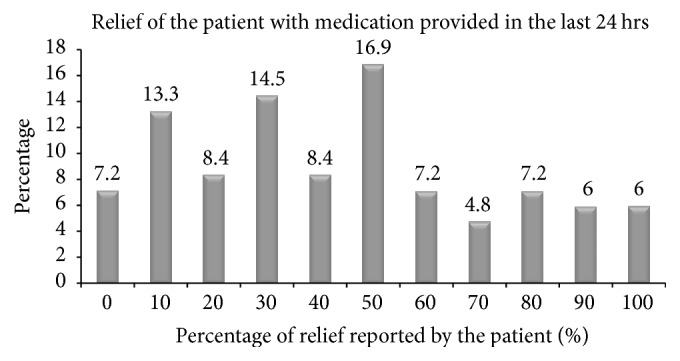
Percentage of pain relief with medications provided in the last 24 hrs, Gondar University Hospital, Gondar, Ethiopia.

**Figure 2:**
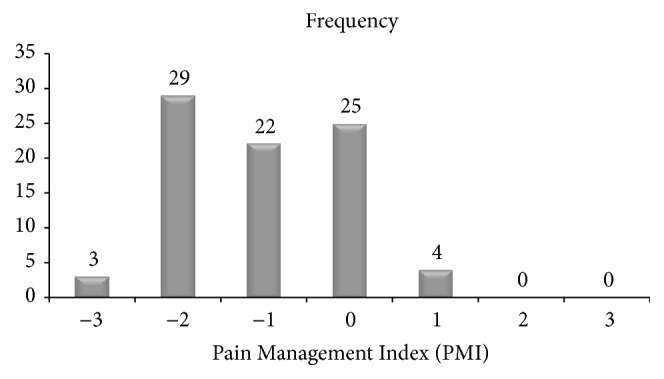
Pain Management Index in cancer patients, Gondar University Hospital, Gondar, Ethiopia.

A total of 76 (91.6%) cancer patients experienced pain with varying degree of severity. Of 83 patients, 7 (8.4%) patients were identified to experience severe pain (Table 2). The severity of pain interference on functioning quality of the cancer patients was also assessed using the multidimensional pain assessment tool (BPI) and 68 of 76 patients with pain (89.2%) experienced interference of pain with functioning. Of 68 patients with pain interference, 41 (53%) patients reported that pain posed moderate to severe interference with their functioning (Table 2). Cancer patients were asked to report their relief to the provided medications and relatively higher proportion of the patients 14/83 (16.9%) have stated that the medications given relieved their pain by 50% (Figure 1). Fifty-four patients (65%) have got their pain undertreated having Pain Management Index (PMI) < 0 whereas 4 (0.05%) patients received overtreatment of analgesia for their pain (PMI = 1) as shown in Figure 2.

**Figure 3:**
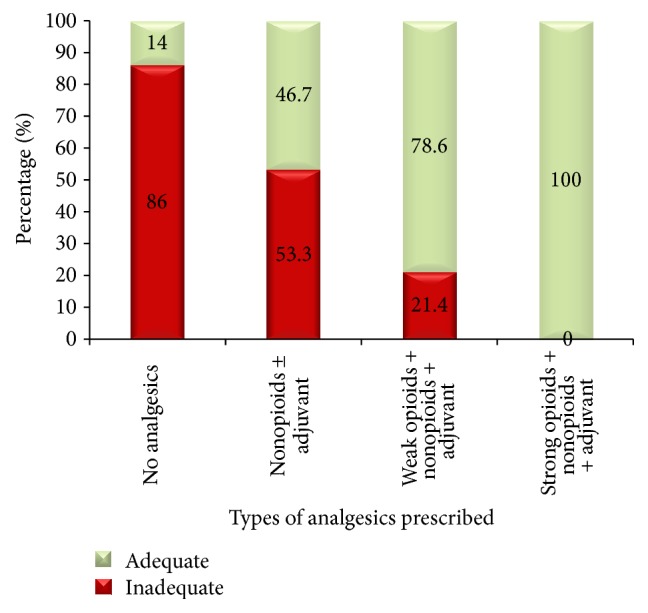
Percentage of cancer pain management adequacy within types of analgesics prescribed, Gondar University Hospital, Gondar, Ethiopia.

Based on the assessment of factors affecting the adequacy of cancer pain management, a higher proportion inadequate pain management 10/13 (76.9%) was observed in cancer patients with high school education level. Cancer pain was not adequately controlled in patients (86%) for whom no analgesics was prescribed (Figure 3).

**Figure 4:**
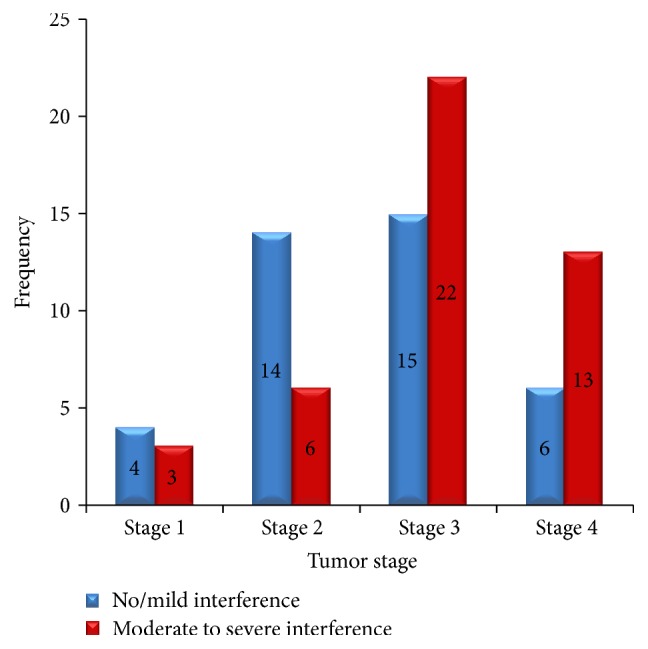
Frequency of pain interference severity within the stage of tumor, Gondar University Hospital, Gondar, Ethiopia.

An assessment of factors affecting the severity of pain interference on functioning was also done. A higher proportion of moderate to severe interference of pain on functioning was observed in cancer patients with stage 3 (59.5%) and stage 4 (68.4%) as shown in Figure 4. Of 29 patients whose pain were adequately treated, mild pain interference on functioning was observed in 10 (34.5%) cancer patients whereas, of 54 patients having inadequately treated pain, 31 (57.4%) patients found their pain to moderately interfere with their functioning.

Upon univariate analysis regarding the adequacy of cancer pain management, COR revealed that pain was more likely to be managed adequately in patients taking nonopioids + adjuvant (COR = 5.4, 95% confidence interval (CI), 1.48–19.55) and weak opioids + nonopioids + adjuvant (COR = 22.5, 95% CI, 4.8–101.5). In addition, number of all medications (COR = 1.78, 95% CI, 1.1–2.76) and number of analgesics (COR = 10.1, 95% CI, 3.66–27.68) had a statistically significant positive association with the likelihood of adequacy of cancer pain management. Patients with comorbidity (COR = 0.2, 95% CI, 0.04–0.80) and Hx of pain (0.13, 95% CI, 0.032–0.53) were less likely to get their pain adequately treated (Table 3). Based on the presence of pain interference on functioning, moderate-severe pain interference was less likely to be present in patients having secondary school educational level (COR = 0.17, 95% CI, 0.032–0.9), cancer patients having tumor stage II (COR = 0.19, 95% CI, 0.051–0.77), and patients whose pain was adequately treated (AOR = 0.39, 95% CI, 0.154–0.98) (Table 4).

Multivariate analysis regarding the adequacy of cancer pain management showed that patients who had attended college/university (AOR = 0.2, 95% CI, 0.07–0.54) and patients with metastasis (AOR = 0.33, 95% CI, 0.12–0.6) were less likely having adequate pain management. An increase in the number of analgesics prescribed was positively associated with adequacy of pain management (AOR = 4.2, 95% CI, 1.87–11.5). Pain was about 9.6 times more likely to be managed adequately in patients prescribed with weak opioids + nonopioids + adjuvant (Table 3). Considering interference of pain on functioning as the outcome of interest, it was found to have statistically significant association with stage of tumor, presence of metastasis, history of treatment modality, history of pain, and pain management adequacy. Cancer patients with tumor stage I (AOR = 0.27, 95% CI, 0.08–0.56) and stage II (AOR = 0.09, 95% CI, 0.03–0.43) and patients adequately treated (AOR = 0.46, 95% CI, 0.065–0.67) were less likely to have pain interference on functioning. Pain was more likely to interfere with the quality of patient functioning in patients with metastasis (AOR = 2.35, 95% CI, 1.46–6.35) and those who had a history of both surgery and chemotherapy treatment (AOR = 3.01, 95% CI, 1.56–7.34). Pain was about 16 times more likely to interfere with functioning in patients who had a history of pain (AOR = 16.45, 95% CI, 1.32–204.69) than those who have never experienced pain before (Table 4).

## 4. Discussion

Pain is a known symptom in patients with cancer interfering on functioning and leading to poor health outcome unless adequately managed. Several studies have examined undertreatment of cancer pain in different setup and populations [2, 4, 6, 13–27]. The aim of this study was to look into the prevalence of inadequacy of cancer pain management and the subsequent pain interference with functioning of cancer patients visiting oncology ward of GUH. No studies have looked at the adequacy of cancer pain management in Ethiopia.

Our study revealed that total of 76 (91.6%) cancer patients experienced pain with varying degree of severity. This finding is higher than other studies: 73.3% in Qadire et al. [13], 34% in Williams et al. [21], and 81.1% in Palalogos et al. [19]. This deviation may be well explained by poor awareness of healthcare providers on pain assessment; clinical practice relying on personal experience; absence of pain assessment and treatment guidelines; and inconsistent availability of pain medications such as opioids. The prevalence of severe pain in this study was found to be 7 (8.4%). Previous studies reported higher percentage of patients with severe pain ranging from 6.2% to 82% [4, 19, 25, 27]. Reporting of patient's pain severity may alert physicians to prescribe analgesics aptly to optimize pain management. In this study, multidimensional pain assessment has been employed including nature and site of the pain occurring to determine the types of cancer pain. Identifying the specific patient's cancer pain type assists health care providers to opt for the right treatment option, thereby improving medication adherence, patient's satisfaction, and health outcome.

Fifty-four (65%) patients were receiving inadequate cancer pain treatment with negative PMI which is higher than those reported by Apolone et al. and Mercadante et al. [4, 27]. However, a review article by Greco et al. reported that inadequate cancer pain treatment can range from 8% to 82% [24]. On the other hand, percentage of inadequate cancer pain treatment can be influenced by the study setting. Two studies reported lower rates of inadequate management of cancer pain in the outpatient setting, 33% [2] and 52.3% [22]. However, a study comparing inadequacy of cancer pain management between outpatient and inpatient settings is needed. Although barriers to cancer pain management such as patient related factors [7, 25] and health professional related factors [28–31] may potentially contribute to the undertreatment of cancer pain, these were not assessed in the present study. However, patients with metastasis and those who have attended college/university were found to be associated with inadequate pain management, whereas, the number of analgesics prescribed and patients prescribed with weak opioids were positively associated with adequacy of pain management in this study. On the contrary, nonadvanced stage of cancer [9], worry about opioid addiction by the patient [25], and lack of adjuvant therapy [4] were identified to be predictors of inadequate cancer pain management by previous studies.

Little is known about the contributing factors of the prevalence of moderate to severe pain interference on functioning. In our study, cancer patients with tumor stage I and stage II (nonadvanced stage) and patients with adequately treated pain were less likely to experience pain interference on functioning. Patients with metastasis and those who had a history of both surgery and chemotherapy treatment and a history of pain were more likely to pose moderate to severe pain interference on functioning.

## 5. Limitations

We could not assess patient's barrier to cancer pain management and healthcare provider's factors such as year of experience of the prescriber as a potential factor for under analgesic treatment. The sample size obtained during the study period is also small. This is a single center study done in GUH as it is the only cancer center in Amhara region.

## 6. Conclusion

Based on the findings of our study, a significant percentage (91.6%) of patients with cancer experience pain of which nearly two-thirds of them (65%) were receiving inadequate cancer treatment and 89.2% of them experienced pain interference with their daily activities. It is also vital to anticipate and assess pain of the cancer patients as routine clinical practice to optimize analgesic therapy through identifying and intervening barriers of adequacy of pain management, thereby improving patient health outcome and quality of life.

## Figures and Tables

**Figure 5:**
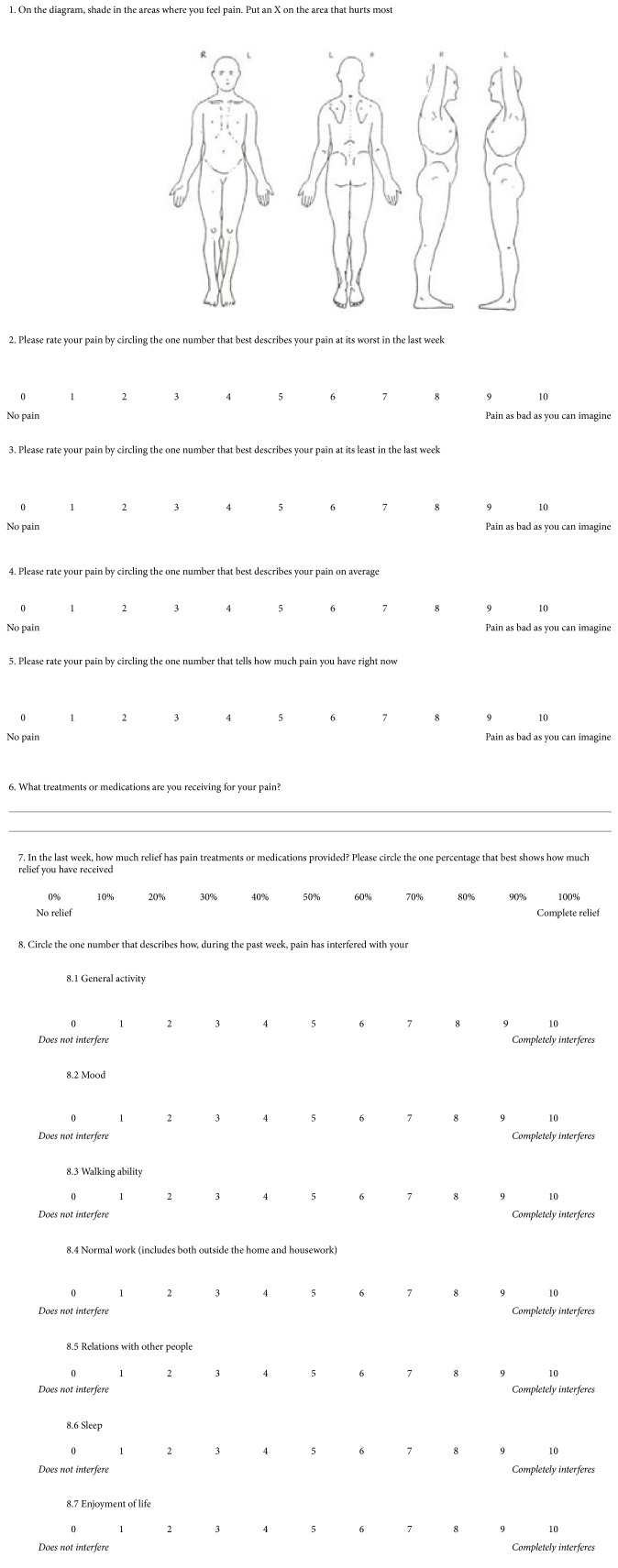
English version of the BPI.

**Figure 6:**
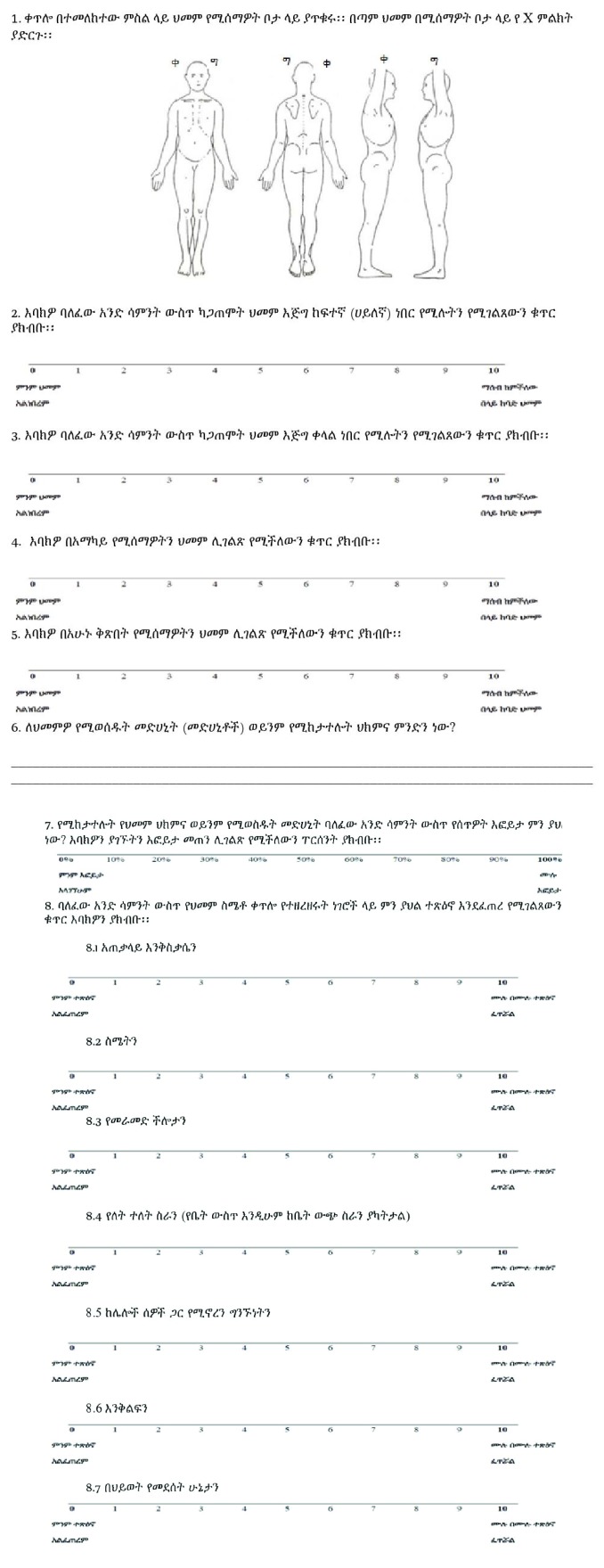
Amharic version of the BPI.

**Table 1:** Characteristics and clinical data of the study population in Oncology Department of Gondar University Hospital, Gondar, Ethiopia.

Demographic and clinical data	Number (%)
Total number of study population, *N*	83
Age in years, median (range)	50 (18–72)
Sex (male)	42 (50.6)
Occupational status	
Student	5 (6)
Government employee	18 (21.7)
Merchant	11 (13.3)
Farmer	33 (39.8)
Private work	12 (14.5)
None	4 (4.8)
Level of education	
Illiterate	42 (50.6)
Primary school	18 (21.7)
Secondary school	13 (15.7)
College/university	10 (12)
Site of cancer	
Genitourinary cancer	21 (25.3)
Gastrointestinal cancer	19 (22.9)
Breast cancer	18 (21.7)
Head and neck cancer	6 (7.2)
Bronchopulmonary cancer	4 (4.8)
Follicular lymphoma	4 (4.8)
Cancer of unknown primary	4 (4.8)
Others	7 (8.4)
Tumor stage	
Stage 1	7 (8.4)
Stage 2	20 (24.1)
Stage 3	37 (44.6)
Stage 4	19 (22.9)
Metastasis (present)	19 (22.9)
Hx of cancer treatment modality	
Chemotherapy	61 (73.5)
Surgery	5 (6)
Combination	17 (20.5)
Comorbidity (present)	16 (19.3)
Number of all medications prescribed, mean ± SD	5.98 (±1.19)
Number of analgesics prescribed, median (range)	0 (0,3)
Types of analgesic	
No analgesics	50 (60.2)
Nonopioids ± adjuvant	15 (18.1)
Weak opioids ± nonopioids ± adjuvant	14 (16.9)
Strong opioids ± nonopioids ± adjuvant	4 (4.8)
Hx of any pain (yes)	71 (85.5)
Types of pain (*N* = 76)	
Nociceptive pain	33 (43.4)
Neuropathic pain	17 (21.7)
Mixed	26 (34.9)

**Table 2:** Frequency of pain severity and pain interference on functioning among cancer patients at Gondar, University Hospital, Gondar, Ethiopia.

Variables	Frequency (percentage)
Pain severity (*N* = 83)	
No pain	7 (8.4)
Mild	21 (25.3)
Moderate	48 (57.8)
Severe	7 (8.4)
Pain interference on functioning (*N* = 76)	
No pain interference	8 (10.8)
Mild	27 (36.2)
Moderate	37 (48.2)
Severe	4 (4.8)

**Table 3:** Relationship between predictive variables and adequacy of cancer pain management at Gondar University Hospital, Gondar, Ethiopia.

Variables	Adequacy of cancer pain management		*X* ^2^, *P* value	AOR (95% CI)	*P* value
	Adequate	Inadequate			
Gender			1.15, 0.2		
Male	17	25		36 (0.36, 3265)	0.12
Female	12	29		1	Rf
Level of education			6.9, 0.069		
Illiterate	15	27		1	Rf
Primary school	4	14		0.54 (0.01, 25.1)	0.73
Secondary school	7	3		0.02 (0.00, 4.13)	0.14
College/university	3	10		**0.2 **(0.07,0.54)^**∗**^	**0.03**
Metastasis			2.85, 0.073		
Present	4	15		**0.33 **(0.12,0.6)^**∗**^	**0.04**
Absent	41	23		1	1
No of all medications (mean)	6.4	5.72	—	0.78 (0.008, 1.77)	0.065
Number of analgesics (mean)	1.03	0.2	—	**4.2 **(1.87,11.5)^**∗∗**^	**0.003**
Types of analgesic			**29.16, **0.00^**∗**^		
No analgesics	7	43		1	Rf
Nonopioids ± adjuvant	7	8		2.3 (0.94, 6.55)	0.21
Weak opioids ± nonopioids ± adjuvant	11	3		**9.6 (4.11, 19.8**)^**∗∗**^	**0.005**
Strong opioids ± nonopioids ± adjuvant	4	0		23 (0.0, NA)	0.76
Comorbidity			**4.39, **0.03^**∗**^		
Present	2	14		0.24 (0.009, 3.22)	0.4
Absent	27	40		1	Rf
Types of pain (*N* = 76)			5.00, 0.08		
Nociceptive pain	5	21		0.439 (0.03, 7.14)	0.56
Neuropathic pain	15	18		6.65 (0.429, 102.9)	0.18
Mixed	6	11		1	Rf
Hx of pain			**9.90, **0.03^**∗**^		
Yes	20	51		0.09 (0.01, 5.11)	0.41
No	9	3		1	Rf

*Notes*. ^**∗∗**^Statistically significant at *P* < 0.01. ^**∗**^Statistically significant at *P* < 0.05; COD, crude odds ratio; AOR, adjusted odds ratio; CI, confidence interval; Rf, reference variable.

**Table 4:** Relationship between predictive variables and severity of pain interference on functioning at Gondar University Hospital, Gondar, Ethiopia.

Variables	Severity of pain interference on functioning		*X* ^2^, *P* value	AOR (95% CI)	*P* value
	Moderate-severe	No/mild			
Age in years (mean)	50.20	47.23	—	1.02 (0.24, 11.22)	0.43
Gender			**7.93, **0.01^**∗**^		
Male	16	26		0.52 (0.56, 7.33)	0.37
Female	28	13		1	Rf
Level of education			7383, 0.22		
Illiterate	25	17		1	Rf
Primary school	9	9		0.59 (0.05, 6.66)	0.47
Secondary school	8	5		1.85 (0.434, 5.23)	0.50
College/university	2	8		0.85 (0.85, 2.23)	0.12
Stage of tumor			9.54, 0.10		
Stage I	3	4		**0.27 **(0.08,0.56)^**∗**^	**0.03**
Stage II	6	14		**0.09 **(0.03,0.43)^**∗**^	**0.02**
Stage III	22	15		0.35 (0.067, 1.46)	0.13
Stage IV	13	6		1	Rf
Metastasis			3.15, 0.18		
Present	11	8		**2.35 (1.46, 6.35**)^**∗∗**^	**0.001**
Absent	26	38		1	Rf
History of treatment modality			6.53, 0.18		
Chemotherapy	28	33		1	Rf
Surgery	3	2		2.7 (0.67, 5.11)	0.38
Combination	13	4		**3.01 **(1.56,7.34)^**∗**^	**0.04**
Comorbidity			2.24, 0.41		
Present	11	5		2.00 (0.8, 12.1)	0.35
Absent	33	34		1	Rf
Hx of pain			**12.53, **0.00^**∗**^		
Present	43	28		**16.45 (1.32, 204.69**)^**∗**^	**0.029**
Absent	1	11		1	Rf
Pain management adequacy			4.92, 0.06		
Present	11	18		**0.46 **(0.065,0.67)^**∗**^	**0.034**
Absent	33	20		1	Rf
PMI (mean)	−1.23	−0.795	—	**0.88 **(0.09,1.01)^**∗**^	**0.06**

*Notes*. ^**∗∗**^Statistically significant at *P* < 0.01. ^**∗**^Statistically significant at *P* < 0.05; COD, crude odds ratio; AOR, adjusted odds ratio; CI, confidence interval; Rf, reference variable.

## Appendix

See Figures 5 and 6.
